# Hemagglutinin expressed by yeast reshapes immune microenvironment and gut microbiota to trigger diverse anti-infection response in infected birds

**DOI:** 10.3389/fimmu.2023.1125190

**Published:** 2023-04-18

**Authors:** Ruyu Xie, Huixia Zhang, Han Zhang, Changyan Li, Daqing Cui, Shujun Li, Zexing Li, Hualei Liu, Jinhai Huang

**Affiliations:** ^1^ School of Life Science, Tianjin University, Tianjin, China; ^2^ China Animal Health and Epidemiology Center, Qingdao, Shandong, China

**Keywords:** influenza A virus, H5N8, *Saccharomyces cerevisiae*, vaccine, hemagglutinin (HA)

## Abstract

**Introduction:**

The H5N8 influenza virus is a highly pathogenic pathogen for poultry and human. Vaccination is the most effective method to control the spread of the virus right now. The traditional inactivated vaccine, though well developed and used widely, is laborious during application and more interests are stimulated in developing alternative approaches.

**Methods:**

In this study, we developed three hemagglutinin (HA) gene-based yeast vaccine. In order to explore the protective efficacy of the vaccines, the gene expression level in the bursa of Fabricius and the structure of intestinal microflora in immunized animals were analyzed by RNA seq and 16SrRNA sequencing, and the regulatory mechanism of yeast vaccine was evaluated.

**Results:**

All of these vaccines elicited the humoral immunity, inhibited viral load in the chicken tissues, and provided partial protective efficacy due to the high dose of the H5N8 virus. Molecular mechanism studies suggested that, compared to the traditional inactivated vaccine, our engineered yeast vaccine reshaped the immune cell microenvironment in bursa of Fabricius to promote the defense and immune responses. Analysis of gut microbiota further suggested that oral administration of engineered ST1814G/H5HA yeast vaccine increased the diversity of gut microbiota and the increasement of Reuteri and Muciniphila might benefit the recovery from influenza virus infection. These results provide strong evidence for further clinical use of these engineered yeast vaccine in poultry.

## Introduction

The H5N8 avian influenza viruses are highly pathogenic threats to poultry and public health (1, 2). The H5N8 subtype AIV has spread globally along with the migration of wild birds and human infection cases have been reported continually during the last decades (3–5). Vaccination is one of the most effective methods to prevent and control the pandemic influenza right now (6, 7). The traditional inactivated vaccine and protein subunit vaccine are widely used vaccines in China (8, 9). The inactivated vaccine for H5N8, although used for decades, has its critical limitations, including a lengthy manufacturing process, the need for lots of fertilized chicken eggs, and a laborious vaccination procedure (10, 11). Moreover, antigen drift is common phenomena in avian influenza H5N8 virus infection (12). The antigenicity changes will reduce the neutralization effect of the inactivated vaccine against the popular virus and the resistance of the vaccine to different subtypes of avian influenza, such as H5N6, has great differences. A safe, convenience and effective vehicle for rapidly developing vaccine against popular subtypes of avian influenza is urgently needed. Viral proteins such as HA have been demonstrated to be administrated as protein subunit vaccine to provide protective efficacy against influenza, with virus vector or yeasts (13). Adenovirus, Newcastle disease virus and fowlpox virus have been reported to be used as vehicles of the influenza protein subunit vaccine for their immunogenicity as virus (14). In this case, reverse genetic manipulation of recombinant virus with proper antigen is a complicated and critical step for successful vaccines (15, 16). Yeast surface display approach has been explored for vaccines against different viruses (17–20). There are several advantages of using yeast as vaccine vehicles (21). First, yeast can be easily modified to express new antigen protein on the surface of yeast or secreted to the culture medium. Second, the oral administration of yeast could stimulate the mucosal-associated immunity in the context of vaccination, as the properties of yeast wall cell composition could promote immune stimulation and the whole cell of yeast could be engulfed by professional antigen-presenting cells due to its diameter. Third, the antigen target protein expressed by yeast could be further processed by glycosylation modification, which may facilitate their recognition by host immune system and enhance the related immune responses. Besides, there are a few reports about yeast vaccine for the prophylactic and therapeutic cases recently. However, the molecular mechanism of protective efficacy provided by the yeast vaccine was not fully understood.

The concept of nucleic acid vaccine is to deliver target antigen DNA or RNA to cells of the immune system (22, 23). Use of mRNA COVID-19 vaccine has been permitted and the immunization programs have been carried out in several countries around the world since 2020 (24). In contrast to the inactivated vaccine and the protein subunit vaccine, nucleic acid vaccine is thought to be safer, cheaper and easily manufactured with stronger specific immune response. Consistent with the idea of nucleic acid vaccine, yeast is widely used in the delivery of functional DNA and mRNA molecules to monocytes and dendritic cells, which indicated yeast as a promising delivery vehicle of DNA and RNA vaccine. But there is no report about its feasibility as nucleic acid vaccine against the H5N8 avian influenza virus until now.

The intestinal microbial community structure of chicken is diverse and the community is composed of different bacteria, methanogenic archaea, fungi and viruses, which play an important role in inducing and regulating host responses to a variety of pathogens, such as AIV (25, 26). It is reported that the use of probiotics to alter the composition of intestinal microflora has a beneficial effect on immunity to influenza virus infection, mainly due to the activation of natural killer (NK) cells in the lung and spleen and the increased expression of various cytokines in the lung (27). Therefore, understanding the changes of chicken intestinal microbiota after immunization is very important to develop effective yeast vaccines to enhance immunity to the virus.

Herein, to further explore the molecular mechanism of yeast vaccine and evaluate the effect of H5HA-based specific yeast vaccine on chicken upon the H5N8 virus challenge, we employed *Saccharomyces cerevisiae* ST1814G strain to develop three types of yeast vaccine, namely, yeast surface display-based H5HA protein subunit vaccine, H5HA gene-based DNA and RNA combined nucleic acid vaccine, and a protein-nucleic acid combined H5HA vaccine. Our experimental results indicated that our engineered yeast vaccine elicited the humoral immunity and provided partial protective efficacy upon high dose of the H5N8 virus infection. Our mechanism studies suggested that engineered yeast vaccine could reshape the immune cell microenvironment and enhance the defense and immune response of *bursa of Fabricus*. On the other hand, the diversity of gut microbiota was increased after oral administration of our engineered ST1814G/H5HA yeast vaccine, which might further reduce the intestinal tract inflammation, protect intestinal barrier and regulate the metabolic processes to benefit for the recovery of chicken from influenza virus infection. Taken together, our experimental results indicate that the yeast vaccine developed in this study have great potentialities in the use of poultry in future.

## Results

### The design of recombinant protein vaccine, nucleic acid vaccine and protein-nucleic acid combined vaccine

**Figure 1:**
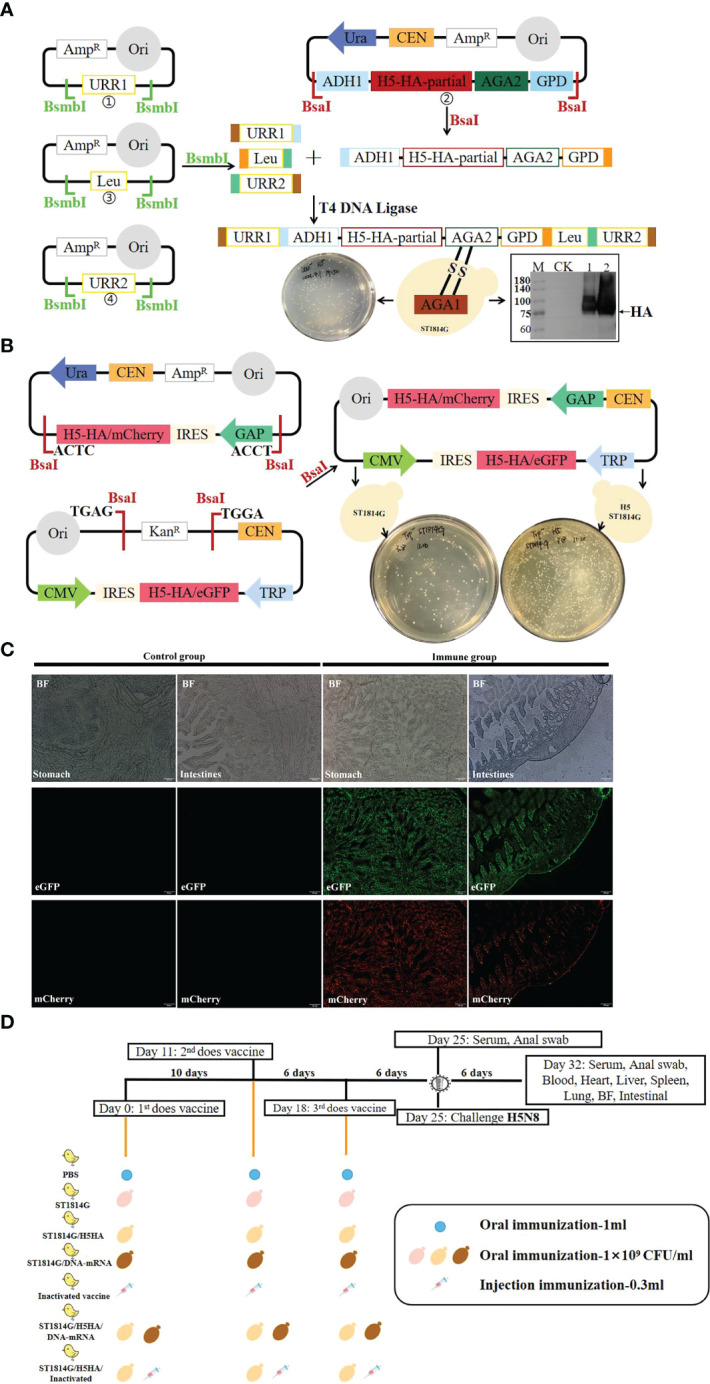
Schematic diagram of vaccines preparation, chicken vaccination and virus challenge experiments. **(A)** Preparation of protein subunit vaccine. The plasmids were constructed as described in Materials and Methods and then transformed into *Saccharomyces cerevisiae* ST1814G strain to prepare ST1814G/H5HA vaccine. Western blot analysis showed that HA protein could be expressed normally in ST1814G/H5HA vaccine. (M: protein marker; CK: ST1814G yeast lysates, lane 1-2: ST1814G/H5HA yeast lysates. **(B)** Preparation of DNA-RNA nucleic acid vaccine. The plasmids were constructed as described in Materials and Methods and then transformed into *Saccharomyces cerevisiae* ST1814G strain or ST1814G/H5HA strain to prepare ST1814G/DNA-RNA vaccine or ST1814G/H5HA/DNA-RNA vaccine. **(C)** Both the eGFP protein and the mCherry protein could be efficiently expressed in stomach and intestine tissues of chickens. **(D)** Seven groups of two-week-old chickens were immunized as the schedule indicated and they were challenged intramuscularly with the H5N8 virus on the 6th day after the third immunization.

We firstly constructed a recombinant S. cerevisiae ST1814G (MATa aga1 his3Δ200 leu2Δ0 lys2Δ0 trp1Δ63 ura3Δ0 met15Δ0) strain expressing 17-484 amino acids of H5HA protein excluding the signaling peptides, which was integrated into the S. cerevisiae chromosome IV according to the methods as our previous studies (28), and the HA protein was expressed correctly as expected as shown in **Figure 1A**. This genetically engineered yeast strain was used as recombinant protein vaccine (ST1814G/H5HA) and it also serves as yeast chassis for our next protein-nucleic acid combined vaccine.

To develop an efficient nucleic acid vaccine system, we constructed a plasmid chassis of DNA and RNA combined nucleic acid vaccine, including a CMV promoter and IRES sequence-driven DNA nucleic acid element and a GAP promoter and IRES sequence-driven mRNA nucleic acid element (**Figure 1B**). The IRES element is supposed to increase levels of translation of recombinant antigen in mammalian cells and prevent translation in yeasts. The CMV promoter and GAP promoter were supposed to promote the transcription of target genes in mammalian cells and yeast, respectively. Based on ST1814G and ST1814G/H5HA yeast strain, we constructed an eGFP-mCherry vaccine (ST1814G/eGFP-mCherry), which was used for the efficacy test of the plasmid chassis, an H5HA-DNA-mRNA vaccine (ST1814G/DNA-mRNA), which was used as nucleic acid vaccines, and an H5HA protein-nucleic acid combined vaccine (ST1814G/H5HA/DNA-mRNA). As shown in **Figure 1C**, both the eGFP and the mCherry proteins could be efficiently expressed in stomach and intestine tissues of chickens. Therefore, the designed antigen, H5HA protein, should be efficiently expressed theoretically both in the ST1814G/DNA-mRNA nucleic acid vaccine and in the ST1814G/H5HA/DNA-mRNA protein-nucleic acid combined vaccine.

### Stronger humoral immunity and efficient protective effect were elicited after vaccination with our engineered yeast vaccine

**Figure 2:**
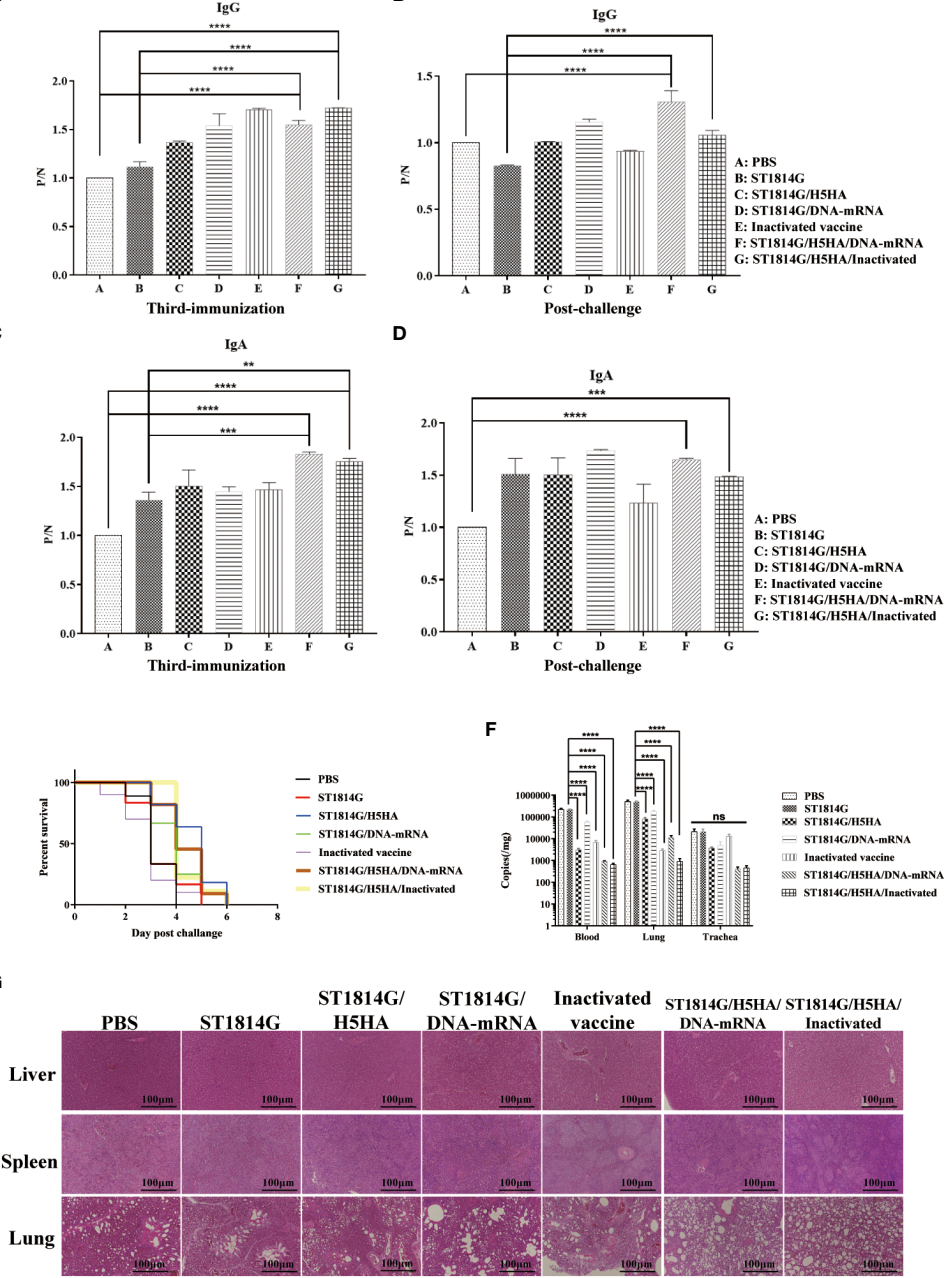
Protective effects of engineered yeast vaccine. **(A–D)** The level of both the secreted IgA in anal swabs and the IgG in serum of each group. The significance of differences was determined by two-way analysis of variance. **(A, C)**, samples after the third immunizations; **(B, D)**, samples post challenge), (**p < 0.01; ***p < 0.001; ****p < 0.001). **(E)** Survival rate of each group at the indicated time post infection. **(F)** Virus titer determination of each group in the blood and lung. Statistical analysis was analyzed by Student’s t-test. (****p < 0.001 n=3 chickens for each group). **(G)** H&E-stained section of the liver and spleen from control group showing spot of bleeding, the lung was showing a pattern of necrotizing alveolitis, the alveolar walls are necrotic and alveolar air spaces contained inflammatory cells in the control groups.

To better understand the efficacy of our engineered yeast vaccine, we next carried out the vaccination procedure and virus challenge experiments as indicated in **Figure 1D** and subsequently, the humoral immune responses were evaluated by ELISA assay as planned. As shown in **Figures 2A–D**, the antibody level of both the secreted IgA in anal swabs and the IgG in serum of the immunized groups, whatever the commercial inactivated vaccine or each of our designed engineered yeast vaccine, were higher than those in PBS treated group or ST1814G yeast treated group before the H5N8 virus challenge. Interestingly, compared to the yeast vaccine alone, our protein-nucleic acid vaccine (ST1814G/H5HA/DNA-mRNA) and recombinant protein vaccine combined with the commercial vaccine (ST1814G/H5HA/Inactivated) exhibited excellent abilities to elicit high levels of IgG and IgA, even after the H5N8 virus challenge. These results indicated our engineered yeast vaccine stimulate the humoral immune responses efficiently against the H5N8 virus infection. Consistent with the humoral immune responses, although it is lethal to the chickens of all the groups with our experimental dose, our yeast-based vaccine could prolong the survival time of infected chickens compared with the PBS treated or ST1814G yeast treated group, which began to die on the second day and over 50 percent of them was dead on the third day (**Figure 2E**).

To further confirm the protective effect of our engineered yeast vaccine, we measured the virus titer in the blood, lung (**Figure 2F**) and trachea (**Supplementary Figure 1A**) of each group. As shown in **Figure 2F**, although combined use could not further decrease the viral load compared with the inactivated vaccine, yeast-based vaccines, both ST1814G/H5HA and ST1814G/DNA-mRNA, were significantly decreased the viral load in blood and lung tissue, which suggested our yeast vaccine could also provide comparable protective efficacy compared with the inactivated vaccine.

The histopathological examination showed that the H5N8 virus infection induced spot of bleeding in liver and spleen, the central vein of liver was congested with lymphocyte infiltration and the boundary between red pulp and white pulp of spleen was not clear, the lung showed a pattern of necrotizing alveolitis, the alveolar walls were necrotic and alveolar air spaces contained inflammatory cells in the control groups (**Figure 2G**). Meanwhile, the immunized groups presented only slight histopathological changes compared with the control groups, indicating that our engineered yeast vaccine could effectively alleviate the histopathological damage caused by the H5N8 infection in chickens.

Collectively, these results demonstrate that our engineered H5HA-based yeast vaccine could elicit the humoral immunity and provide protective efficacy for chickens after the H5N8 virus infection and we found that simultaneous administration of different types vaccines may be a better choice for the vaccination procedure.

### The immune cell microenvironment of *bursa of Fabriciu* was reshaped to enhance the defense and immune response

**Figure 3:**
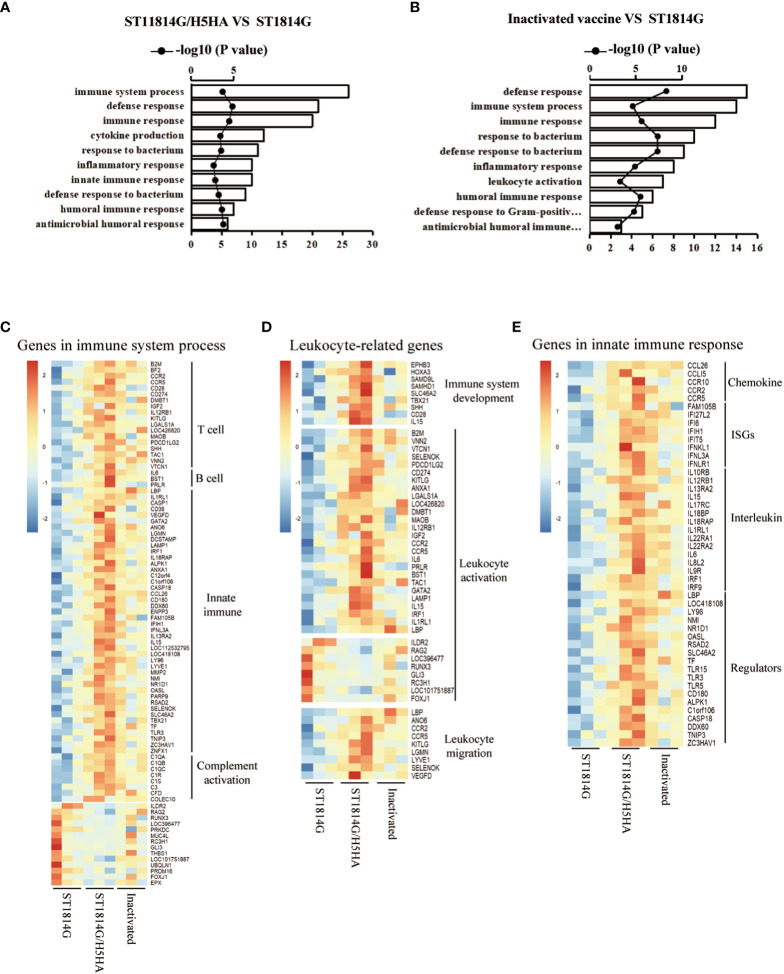
Effect of Vaccines on immune related genes of *bursa of Fabricius* in chickens. **(A, B)** Biological process analysis of the differentially-expressed genes (DEGs) based on the Gene Ontology databases. Abscissa: biological process; Ordinate: number of genes and –log10 (P value); **(A)** ST1814G/H5HA group vs ST1814G group; **(B)** Inactivated vaccine group vs ST1814G group. **(C–E)** Heatmaps of gene expression levels in BF for indicated gene lists. **(C)** Genes related to immune system process; **(D)** Genes related to leukocyte microenvironment of *bursa of Fabricius*; **(E)** Genes related to innate immune response.

Yeasts have been considered as a promising delivery platform for recombinant protein and nucleic acid vaccines for decades. To better understand the molecular mechanisms of the protective efficacy after immunized with yeast vehicles and the traditional inactivated vaccines, we next performed RNA sequencing using the *bursa of Fabricius*(BF) of chickens and the differentially-expressed genes (DEGs) were proceeded to enrichment analysis based on the Gene Ontology databases. With regards to our engineered yeast vaccine, lots of defense-related genes were significantly upregulated in chickens immunized with ST1814G/H5HA vaccine compared with those in the ST1814G yeast placebo (**Figures 3A**). The related biological processes included defense response, immune response and response to biotic stimulus, which might be responsible for the protective efficacy of our engineered yeast vaccine in chickens upon the H5N8 virus infection. For the traditional inactivated vaccine, enrichment analysis of upregulated genes also indicated that defense-related biological processes were activated in these chickens (**Figure 3B**). To further verify the influence of engineered yeast vaccine on the immune system of chicken, we next checked and verified the gene expression levels of immune system process (**Supplementary Figures 1B, D**). We found that, compared with the ST1814G group and the inactivated vaccine group, there were about 73 genes upregulated significantly, which play roles on positive regulation of the immune system process, and about 14 genes downregulated significantly, which play roles on negative regulation of the immune system process in the ST1814G/H5HA groups (**Figure 3C**).

The *bursa of Fabricius* is an important organ of chicken for the immune cell differentiation and proliferation and the microenvironments are crucial factors for the immune cell determination. As the immune system processes were significantly different in our engineered yeast vaccine group, we next checked the expression levels of leukocyte type-related genes to generally evaluate the change of the microenvironment of *bursa of Fabricius*. As shown in **Figure 3D**, compared with the ST1814G group and the inactivated vaccine group, the expression levels of genes regulating the immune system development and leukocyte migration were upregulated significantly in ST1814G/H5HA group. At the same time, the expression levels of genes, which were reported to positively regulate of lymphocyte, such as T cell and B cell, were also increased in ST1814G/H5HA group compared with the ST1814G group or the inactivated vaccine group. Additionally, the expression levels of genes in innate immune response, such as the interleukin receptors (ILRs), interferon ligands (IFNLs) and C-C Motif Chemokines receptors (CCLRs), which are important for the lymphocyte function, were also increased in the ST1814G/H5HA group (**Figure 3E**). These results suggested that the immune cell types produced in the *bursa of Fabricius* of ST1814G/H5HA group might be different compared with the ST1814G group and the inactivated vaccine group. Collectively, our above results indicated that the stimulation with our yeast-based influenza vaccine might reshape the immune cell microenvironment and it would elicit much more immune and defense responses compared with the traditional inactivated vaccine in chickens.

### Yeast vaccine elevates the diversity of gut microbiota that might benefit the recovery from influenza virus infection

**Figure 4:**
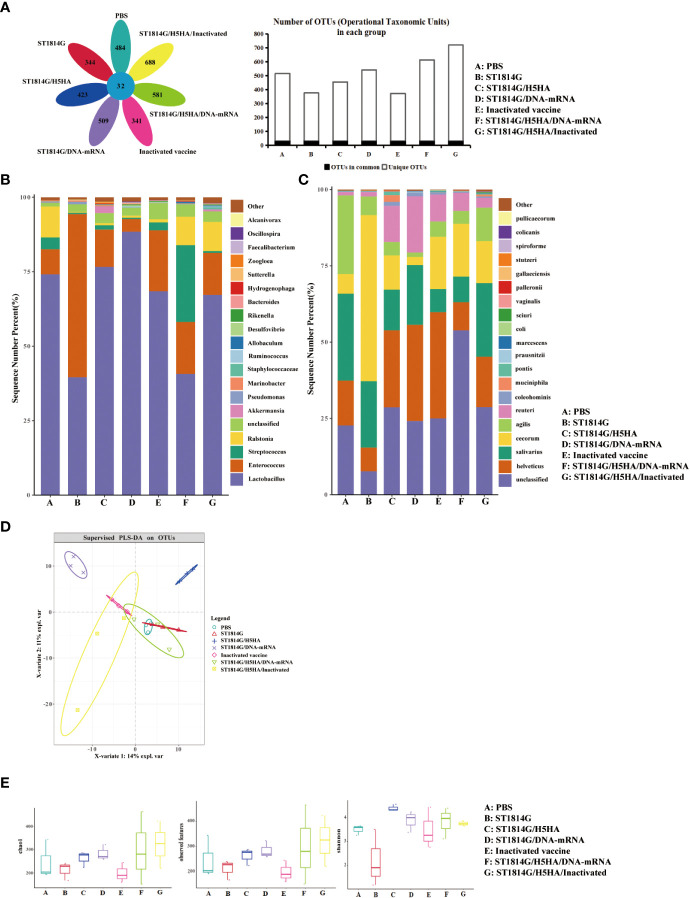
Effect of vaccines on intestinal microflora diversity post immunization. **(A)** Venn diagrams (left) and bar charts (right) of common or endemic species. **(B)** Relative content of bacterial genus in each group. **(C)** Relative content of bacterial species in each group. **(D)** Partial least-squares discrimination analysis (PLS-DA) revealed the diversity of chicken gut microbiota among the seven groups. Dots represent individual samples. **(E)** The analysis of Alpha diversity index, Chao1, Shannon and observed features, showed the diversity of chicken gut microbiota within each group post immunization. Dots represent individual samples.

Microorganisms play important roles in the regulation of gut micro-ecology balance, especially during influenza virus infection. To better understand the molecular mechanism of the protective effect on the gut microbiota, we next performed 16S rRNA sequencing and investigated the diversity of gut microbiota in different groups after the H5N8 virus challenge. As shown in **Figure 4A**, the results indicated that there were much more species special sequences in the ST1814G/H5HA/DNA-RNA group and the ST1814G/H5HA/Inactivated group than the PBS group or the ST1814G group while the inactivated vaccine group did not show this advantage.

Taxonomic analysis of the gut microbiota was performed to further explore the composition differences of species and genera in each group. As shown in **Figures 4B, C**, at genus level, the relative content of *Lactobacillus* was significantly higher in the ST1814G/DNA-RNA groups compared to the ST1814G group. *Lactobacillus* are supposed as probiotic bacteria (29), which plays roles on regulating gut microbiota, enhancing mucosal immune, and protecting gastric mucosa. At the same time, the relative content of *Akkermansia* increased significantly in ST1814G/H5HA group and ST1814G/H5HA/Inactivated group. Usually, *Akkermansia* is a marker of better growth status and diversity of gut microbiota. The difference with them is that the proportion of *streptococcus* in group ST1814G/H5HA/DNA-RNA was significantly increased. *Streptococcus* had critical contributions to the maintenance of intestinal homeostasis (30). This suggested that different combinations of vaccines affect the distribution of bacterial flora in the gut. At species level, the two species with large changes were *Reuteri* and *Muciniphila* (31–34). The relative content of *Reuteri* was significantly higher in the immunized groups, especially in the ST1814G/DNA-RNA group. *Reuteri* was reported to reshape the gut microbiota to reduce inflammation (35–37). The relative content of *Muciniphila* was higher in ST1814G/H5HA group and ST1814G/H5HA/Inactivated group, the combination of ST1814G/H5HA and inactivated vaccine led to a decrease in the proportion of *Muciniphila* but increased the number of microbiomes in the gut. *Muciniphila* is belived to play a protective role in the intestinal tract, as the decrease of *Muciniphila* could cause intestinal barrier damage, plasma endotoxin increase, low-grade inflammation and metabolic disorders (38). These results suggested that our yeast vaccines could improve the probiotic bacteria content of vaccinated chickens, such as *Muciniphila*, which might improve the microbiome in the gut together.

Partial least-squares discrimination analysis (PLS-DA) also showed that the distribution of bacterial communities in the ST1814G/H5HA group was different from the PBS group and the ST1814G group (**Figure 4D**), which were in accord with the increased relative abundance of *Muciniphila* as shown above. The alpha diversity analysis of each group showed that Shannon index was higher in the immunized groups, especially in the ST1814G/H5HA group, compared to the PBS group or the ST1814G group (**Figure 4E**), indicating that our engineered yeast vaccine could increase the gut microbiota diversity during influenza virus infection, which might be benefit for the recovery of the infected chickens.

## Discussion

Vaccinations are the most effective manners to prevent the outbreak of avian influenza virus, such as the H5N8 virus. Although the traditional inactivated influenza vaccines are the most popular candidates in China right now, there are several reports about exploring yeast surface-displayed influenza vaccines during the past decades (39), which might be promising vaccine that greatly facilitate the vaccination procedure in poultry. However, the molecular mechanism of protective efficacy provided by the yeast vaccine was not fully understood.

In this study, we developed three yeast-based vaccines: yeast surface-displayed recombinant protein vaccine (ST1814G/H5HA), yeast-based DNA-RNA vaccine (ST1814G/DNA-RNA) and yeast-based protein-nucleic acid combined vaccine (ST1814G/H5HA/DNA-RNA). The vaccination program was designed to test the protective efficacy with the above vaccine alone or with the traditional inactivated vaccine together, which was aimed to explore better vaccine candidate. The results suggested that all of our engineered yeast vaccine could promote the humoral immune response and decrease the viral load in different tissues after high dose of the H5N8 virus challenge. Considering the special antibody response and the viral load experiments, simultaneous administration of ST1814G/H5HA/DNA-RNA or ST1814G/H5HA/Inactivated vaccines may be better candidates for the vaccination procedure in poultry. Unfortunately, the chicken survival experiments did not get a fully protective effect in the simultaneous administration groups or any other group, and it was highly possible that the H5N8 influenza virus was extremely pathogenic for the experiment chickens. Even so, the immunized groups survived longer than the PBS group or the ST1814G group, suggesting that our engineered yeast vaccine offered partial protective effects.

To better understand the molecular mechanism of yeast vaccine, we analyzed the gene expression patterns using the *bursa of Fabricius*, which is an important immune organ of chickens. The results suggested that our ST1814G/H5HA vaccine group greatly enhanced the defense and immune responses and further analysis suggested that the immune cell microenvironment of *bursa of Fabricius* was reshaped after our engineered yeast vaccination in chicken. The genes for immune system development, lymphocytes activation and leukocyte migration were partially upregulated in the ST1814G/H5HA group compared to the PBS group or the inactivated vaccine group. Meanwhile, the genes for innate immune response were also partially upregulated in the ST1814G/H5HA group. These results together suggested that the yeast vaccine not only stimulate the adaptive immune response, but also stimulate the innate immune response in *bursa of Fabricius*. The cross talks between innate immunity and adaptive immunity are extremely important for efficient protection upon pathogen challenge. Taken together, the complexity and crosstalk between the innate and adaptive immune system, stimulated by our engineered yeast vaccine but not the traditional inactivated vaccine, might explain how the yeast vaccine provided protective efficacy *in vivo*.

Gut microbiota have been supposed to provide the host with several functions that promote immune homeostasis, immune responses, and protection against pathogen colonization (40–42). To investigate the influence of our engineered yeast vaccine on the diversity of gut microbiota, we performed the taxonomic and alpha diversity analysis and the results suggested that yeast vaccine elevated the diversity of gut microbiota. The increased content of symbiotic bacterium, such as *Reuteri* which was in enhancing antiviral activity of chicken macrophages against AIV in chicken Macrophages and *Muciniphila*, previous research has shown that oral administration of *A.muciniphila* could decrease the levels of IL-6 and IL-1β in the lungs and the levels of IL-6 and TNF-α in the blood during the course of H7N9 infection, which suggested that inhibition of these cytokines was one of the mechanisms by *A.muciniphila* protects the host from influenza virus infection (43). Therefore, oral administration of our engineered yeast vaccine might use similar mechanisms to protect chickens from AIV.

## Conclusion

We developed three types of the H5N8 HA-based yeast-produced avian influenza vaccines. Our experimental results demonstrated that all of the engineered yeast vaccine elicited humoral immunity in chicken, decreased the virus load in different tissues, and provided partial protection of chickens from high dose of lethal the H5N8 virus infection. It’s just that the combination of vaccines (ST1814G/H5HA/DNA-mRNA and ST1814G/H5HA/Inactivated) compared with yeast vaccine along could enhance more humoral immunity and obviously reduce viral titers in the blood and lungs. Mechanically, yeast vaccine promoted the defense and immune responses, reshaped the immune cell microenvironment in *bursa of Fabricius*. Although the proportion of probiotics in intestinal flora is different between yeast vaccine in separate and combined use, but all yeast vaccine promoted the immune homeostasis upon influenza virus infection. These yeast vaccines can be further used as influenza vaccine for preventing influenza outbreaks in poultry.

## Materials and methods

### Ethics statement

All animal studies were carried out in strict accordance with the recommendations in the Guide for the Care and Use of Laboratory Animals of the Ministry of Science and Technology of the People’s Republic of China. The animal study was reviewed and approved by the Institutional Animal Ethical Committee of Tianjin Institute of Pharmaceutical Research [Certificate Number: SYXK (Jin) 2016-0009, Tianjin, China].

### Preparation of recombinant HA protein

The 97-1131 nucleotides of the H5N8 HA gene were inserted into the restriction site *B amHI* of the pET-28a plasmid vector. The primers used in the study was showed in **Table 1**. The plasmid was transfected into Rossatta *E.coli* cell to induce the expression of HA protein by adding isopropyl-β-D-thiogalactoside (IPTG). The recombinant HA protein was further purified with Ni-NTA agarose beads (Thermo Scientific, 78605).

**Table 1:** PCR primers for expressing vectors.

Primer name	Primer sequence (5′- 3′)
pGPD-Aga2-ADH1-H5HA-F	ACGATAAGGTACCAGGATCCGATCAGATTTGCATTGGTTACCAT
pGPD-Aga2-ADH1-H5HA-R	ACTGTGCTGGATATCTACTGGATCCTTCGAAACAGCCGTTACCCA
CMV-IRES-H5HA-F	AAAACACGATGATAACTCGAGATGGAGAACATAGTACTTCTTCTTGCA
CMV-IRES-H5HA-R	AATTCGAAGCTTGAGCTCGAGTTAAATGCAAATTCTGCACTGTAACG
GAP-IRES-H5HA-F	AAAACACGATGATAACTCGAGATGGAGAACATAGTACTTCTTCTTGCA
GAP-IRES-H5HA-R	AATTCGAAGCTTGAGCTCGAGTTAAATGCAAATTCTGCACTGTAACG
CMV-IRES-eGFP-F	AAAACACGATGATAACTCGAGATGGTGAGCAAGGGCGAGG
CMV-IRES-eGFP-R	AATTCGAAGCTTGAGCTCGAGCTTGTACAGCTCGTCCATGCC
GAP-IRES-mChrry-F	AAAACACGATGATAACTCGAGATGGTGAGCAAGGGCGAGG
GAP-IRES-mChrry-R	AATTCGAAGCTTGAGCTCGAGCTTGTACAGCTCGTCCATGCC
pET-28a-H5HA-F	CAGCAAATGGGTCGCGGATCCCATGCAAACAATTCGACAGAGC
pET-28a-H5HA-R	ACGGAGCTCGAATTCGGATCCCCCATACCAACCATCAACCATT

### Vaccine preparation

#### Preparation of protein subunit vaccine

The 97-1131 nucleotides of the the H5N8 HA gene were inserted into the pGPD-AGA2-ADH1-POT plasmid vector. The plasmids, with terminal homologous arms as indicated, PMV-URR1, PMV-URR2, PMV-Leu and pGPD-AGA2-H5HA-ADH1-POT were digested with restriction enzymes *BsmbI* or *BsaI*, and then the fragments were ligated by T4 DNA ligase. The ligated products were transformed into *Saccharomyces cerevisiae ST1814G strain* (MATa aga1 his3Δ200 leu2Δ0 lys2Δ0 trp1Δ63 ura3Δ0 met15Δ0) and the positive clones were screened on SD/-Leu plate as previous described (44) (**Figure 1A**).

#### Preparation of nucleic acid vaccine

The 1-1704 nucleotides of the H5N8 HA gene were inserted into the pGAP-IRES plasmid vector and pCMV-IRES plasmid vector, respectively. The GAP promoter promotes the transcription of target genes in yeast and the IRES element prevents the translation in yeast, which function together as an RNA production combination. The CMV promoter promotes the transcription of target genes in mammalian cells and the IRES element increases the translation in mammalian cells, which function together as an DNA production combination. The plasmids pGAP-IRES-H5HA and pCMV-IRES-H5HA were digested with restriction enzyme *BsaI* and then the fragments were ligated by T4 DNA ligase. The ligated products were transformed into *Saccharomyces cerevisiae ST1814G strain* (MATa aga1 his3Δ200 leu2Δ0 lys2Δ0 trp1Δ63 ura3Δ0 met15Δ0) as previous described and the positive clones were screened on SD/-Trp plate (**Figure 1B**). For the eGFP-mCherry nucleic acid vaccine, the H5HA gene was replaced by eGFP or mCherry, respectively.

### Inactivated vaccine

The commercial inactivated vaccine used in this experiment was Reassortant Avian Influenza Virus (H5+H7)Trivalent Vaccine (DaHuaNong, 19002346).

### Chicken vaccination and virus challenge experiments

91 of 2-week-old chickens were randomly divided into 7 groups for the indicated vaccination program as **Figure 1D**. Serum samples, anal swab and intestinal contents were collected at the indicated time as the schedule indicated. Chickens were challenged with 10^6.8^ TCID50/0.1 mL the H5N8 influenza virus at the 6th day after the third vaccination and the survival rate was recorded daily. 6 days after the challenge, blood, lung and trachea were collected to measure the viral RNA load. At the same time, the tissues of liver, spleen, lung and BF were prepared for tissue sections.

### Enzyme-linked immunosorbent assay

Purified the H5N8 HA proteins were coated onto 96-well Maxisorp clear plates at 1 mg/mL in 50 mM Na_2_CO_3_, pH 9.6, overnight at 4°C. 500μL serum or PBS buffer-diluted anal swabs were incubated at 37°C for 40 minutes. Plates were washed 3 times with PBST (0.5% Tween-20), and then incubated with HRP-goat anti-chicken IgG (Solarbio, Beijing, China) or goat anti-chicken IgA (Abcam, USA). After 40 minutes incubation at 37°C, plates were washed 3 times with PBST (0.5% Tween-20) and 1-Step Ultra TMB-ELISA (Solarbio, Beijing, China) was added. Following 12 minutes incubation, reactions were stopped with 2 M sulfuric acid. The absorbance of each well at 405 nm was determined using ELISA Reader (Biored, USA).

### Quantitative real-time PCR

Total RNA was extracted with TRIzol reagent (Yeasen Biotech, Shanghai, China). First-strand cDNA was synthesized from 1 μg total RNA using the Hifair®III 1st Strand cDNA Synthesis SuperMix (Yeasen Biotech, Shanghai, China), and qPCR was performed using the Hieff® qPCR SYBR Green Master Mix (Yeasen Biotech, Shanghai, China). The primers used in the study was showed in **Table 2**. The total volume of qPCR was 20μL, and the amplification step was 94 °C for 5 minutes, followed by 40 cycles of denaturation at 94°C for 10 seconds and extension at 60°C for 30 seconds, followed by dissociation curve analysis. ABI 7500 real-time PCR was used for numerical determination, and each sample was analyzed in triplicate. Data were calculated based on the 2^−ΔΔCT^ method. Relative mRNA expression was normalized to that of β-actin.

**Table 2:** qPCR Primers to verify the level of gene expression.

Primer name	Primer sequence (5′- 3′)
IL6-F	CCTGTTCGCCTTTCAGACCT
IL6-R	GGGATGACCACTTCATCGGG
IL15-F	TTGCTCCATAGGTTTCCGAGG
IL15-R	TGTGTTTTCTGACTCTCCGGC
IL10RB-F	GAGCAGACCACCCATAACGG
IL10RB-R	TGAACCACTAGGAGGCTTGC
CCR2-F	GTGTCATGTCAGCACTTTTATGC
CCR2-R	AGCAGCACTGTTCGCCTATG
CCR5-F	CAACGCAACCCATCCTAACAC
CCR5-R	GGCGTAAAACTGTGACAAGC
CCL15-F	CCTGACAAGTGCTGCTTCAAC
CCL15-R	GCTGGTACCTCTTCACCCAC
IFI6-F	AATGGGTGCCAAAGGCTCAA
IFI6-R	TGGACCGCTGCTTCTTTCTA
IFIT5-F	GCACATAAAAGGCAACCCACA
IFIT5-R	ACATCCTCCTTCAGCAAAGTCC
IFIH1-F	GAAGAGGAGAGGAGGCCCAA
IFIH1-R	CTGTCAACCCACACTGGACA

### RNA-seq and 16S rRNA seq

At the 6th day after the third vaccination, three immunized chickens were randomly selected in each group. The total RNA of *bursa of Fabricius* and the segments about 10centimeters from the cecum were sent to RNA-seq and 16S rRNA-seq, respectively.

In RNA-seq analysis, the raw fastq files were analyzed using FastQC (v0.11.9). Sequencing adaptors and low quality (base quality score<20) reads were trimmed by trim galore (v0.6.6). Reads shorter than 20nt were discarded. The trimmed reads were aligned to the *Gallus* (chicken) (GCF_016699485.2) by Hisat2 (v2.2.1) and the aligned data was further proceeded by Samtools (v1.3.1). The transcript was quantified by String Tie (v2.1.7). The gene counts were next analyzed by DESeq2 and the significant different genes were defined as absolute(log2FoldChange) >1.5 and pvalue < 0.05.

In the 16S rRNA analysis, the original sequencing data was filtered firstly, and the processed data was filtered to get effective data. Operational Taxonomic Units (OTU) clustering/denoising and species classification analysis were carried out based on the effective data to form the species abundance spectrum of OTU and other species classification levels. Based on the species abundance spectrum of OTU after homogenization of the data, the abundance and diversity index of OTU were analyzed, and the community structure of species annotation was statistically analyzed at each classification level.

## Data availability statement

The data presented in the study are deposited in the GEO database, accession number GSE220714.

## Ethics statement

The animal study was reviewed and approved by Institutional Animal Ethical Committee of Tianjin Institute of Pharmaceutical Research [Certificate Number: SYXK (Jin) 2016-0009, Tianjin, China].

## Author contributions

Conceived and designed the experiments: JH, HL, RX, HuZ. Performed the experiments: RX, HuZ, HaZ, CL, DC, SL, ZL, HL. Analyzed the data: RX, HaZ, ZL, JH. Wrote the paper: JH, ZL, RX. All authors contributed to the article and approved the submitted version.

## Supplementary material

Supplementary Figure 1 Virus titer determination of each group in the trachea and Verification experiment of gene expression level in the *Bursae of Fabricius* post immunization. **(A)** Virus titer determination of each group in the trachea. Statistical analysis was analyzed by Student’s t-test. (ns, no significance. n=3 chickens for each group). The transcriptional levels of interleukin receptors (ILRs) **(B)**, C-C motif chemokine receptors (CCLRs) **(C)**, and interferon ligands (IFNLs) genes**(D)** in the *Bursae of Fabricius* of the immunized chickens were determined by RT-qPCR. Data were calculated based on the 2−ΔΔCT method, and relative mRNA expression was normalized to that of β-actin. The data in figures were obtained from three independent experiments and represent the averages ± SD. The significance of differences was determined by two-way analysis of variance (*, p < 0.05; **, p < 0.01; ***, p < 0.001; ****, p < 0.001 or ns, no significance).
