# Supplementary figures and images for: Hemagglutinin expressed by yeast reshapes immune microenvironment and gut microbiota to trigger diverse anti-infection response in infected birds
Source: Front Immunol. 2023 Apr 18;14:1125190. doi: 10.3389/fimmu.2023.1125190 (PMC10151582; doi:10.3389/fimmu.2023.1125190)

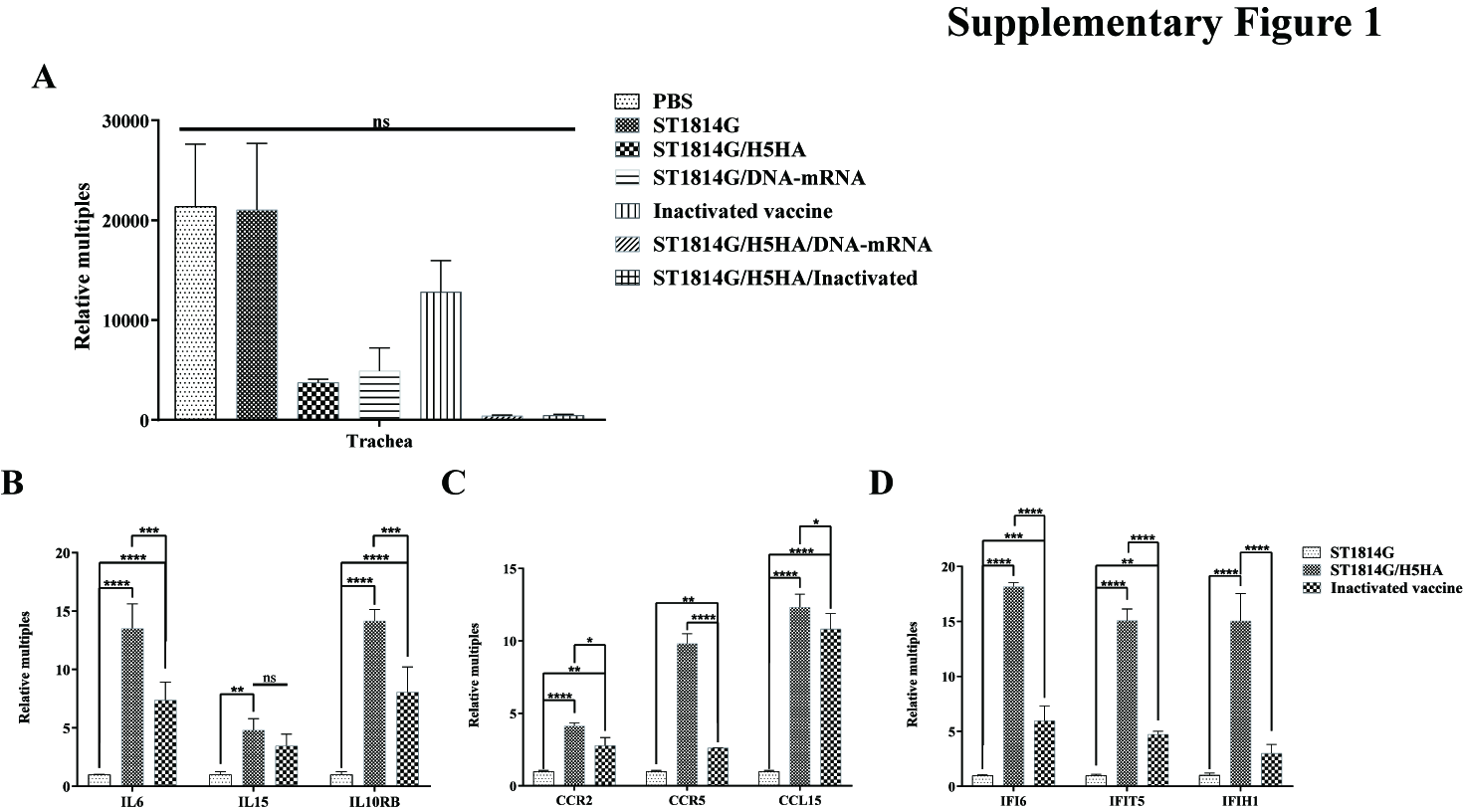

Supplement: Supplementary Figure 1 — Virus titer determination of each group in the trachea and Verification experiment of gene expression level in the Bursae of Fabricius post immunization. (A) Virus titer determination of each group in the trachea. Statistical analysis was analyzed by Student’s t-test. (ns, no significance. n=3 chickens for each group). The transcriptional levels of interleukin receptors (ILRs) (B), C-C motif chemokine receptors (CCLRs) (C), and interferon ligands (IFNLs) genes(D) in the Bursae of Fabricius of the immunized chickens were determined by RT-qPCR. Data were calculated based on the 2−ΔΔCT method, and relative mRNA expression was normalized to that of β-actin. The data in figures were obtained from three independent experiments and represent the averages ± SD. The significance of differences was determined by two-way analysis of variance (*, p < 0.05; **, p < 0.01; ***, p < 0.001; ****, p < 0.001 or ns, no significance). [file Image_1.tif]
